# Tératome immature de l’ovaire: à propos d’un cas

**DOI:** 10.11604/pamj.2017.27.263.6400

**Published:** 2017-08-09

**Authors:** Wail Bouzoubaa, Sofia Jayi, Fatima Zohra Fdili Alaoui, Hikmat Chaara, Moulay Abdelilah Melhouf

**Affiliations:** 1 Service de Gynécologie Obstétrique II, CHU Hassan II, Fès, Maroc

**Keywords:** Tératome ovarien immature, grades, chirurgie conservatrice, Immature ovarian teratoma, grades, conservative surgery

## Abstract

Les tératomes ovariens sont des tumeurs issues des cellules germinales pluripotentes, on décrit 3 types de tératomes: tératome mature, immature et monodermique. Le tératome immature constitue moins de 1% des cancers ovariens, et touche préférentiellement les sujets jeunes. Nous rapportons le cas d'une patiente de 25 ans, admise pour prise en charge d'une masse abdomino-pelvienne pour laquelle elle a bénéficiée d'une échographie et d'un scanner pelvien suivie d'un traitement conservateur a base d'une annexectomie gauche, avec multiples biopsies, dont le résultat anatomo-pathologique était en faveur d'un tératome ovarien immature. Par la suite le traitement a été complété par une hystérectomie avec curage lombo-aortique et omentectomie. Nous insistons à travers cette observation et sous la lumière d'une revue de la littérature sur les terrains particuliers prédisposant à ce type de tumeur rare et grave et sur les différents critères radiologiques orientant vers ce type histologique rare des tumeurs ovariennes, afin d'améliorer le pronostic et la prise en charge de cette pathologie qui reste multidisciplinaires.

## Abstract (en)

Ovarian teratomas are tumors resulting from pluripotent germ cells. We here describe 3 different types of teratomas: mature, immature and monodermal teratomas. Immature teratoma accounts for less than 1% of ovarian cancers and mainly affects young subjects. We report the case of a 25 year female patient, admitted with abdomino pelvic mass. She underwent ultrasound and a pelvic CT scan followed by conservative treatment based on left annexectomy associated with multiple biopsies. Anatomopathological examination showed immature ovarian teratoma. Patient’s treatment was supplemented by a hysterectomy with lumbo-aortic curage and omentectomy. We highlight, through this study and review of the literature, the predisposing factors for this type of rare and severe tumor and the radiological features suggesting these rare histological types of ovarian tumors, in order to improve the prognosis and multidisciplinary management of patients.

## Introduction

Le tératome immature est une tumeur germinale non séminomateuse qui a été décrite pour la première fois en 1960 par Thürlbeck et Scully [1], et qui représente moins d'un pour cent des tumeurs malignes de l'ovaire. Elle touche préférentiellement l'enfant et l'adolescente. Leur classification en trois grades de malignité permet de mieux définir les indications thérapeutiques [2]. À partir de l'histoire clinique d'une patiente de 25 ans prise en charge au service de Gynécologie et Obstétrique II du CHU Hassan II de Fès, pour un tératome immature de l'ovaire, il nous a paru intéressant de rappeler les difficultés diagnostiques, les caractéristiques cliniques et paracliniques, le pronostic de ce type de tumeur de l'ovaire et d'en discuter les modalités de prise en charge.

## Patient et observation

Il s'agit de H.B, âgée de 25 ans, G2P1 (1 enfants vivants), ayant comme antécédents une grossesse extra utérine pour laquelle elle a bénéficiée d'une salpingéctomie droite il y a 1 an, connue porteuse d'un kyste de l'ovaire sous traitement symptomatique a base d'antispasmodiques. Admise dans notre formation pour prise en charge d'une douleur pelvienne latéralisée à gauche, sans symptomatologie gynécologique. Chez qui l'examen a trouvé une patiente stable sur le plan hémodynamique, avec une sensibilité latéro-utérine gauche au toucher vaginal, et la perception d'une masse a travers le cul de sac postérieur. Une échographie pelvienne a objectivée un utérus de taille normale avec la présence en sus utérin d'une image solido-kystique avec cloison mesurant 11/7 cm de grand axe. Un scanner pelvien a confirmé la présence d'une masse pelvienne solidokystique mesurant 110/80 mm, prolabée dans le cul de sac de douglas, rehaussée âpres injection du produit de contraste de façon hétérogène évoquant une masse ovarienne suspecte de malignité. La patiente a bénéficiée d'une laparotomie avec a l'exploration présence d'une masse ovarienne de 11 cm, d'où la réalisation d'une annexectomie gauche avec biopsies multiples (Gouttières parieto-coliques droite et gauche, l'espace vésico-uterin, grand epiploon et le douglas), dont le résultat anatomo-pathologique était un tératome ovarien immature grade 3, avec un tissue graisseux sensiblement normal dans les différentes biopsies réalisés. Apres le résultat anatomopathologique, le geste chirurgical a été complété par la réalisation d'une hystérectomie avec omentectomie et curage pelvien dont le résultat anatomo-pathologique était sans particularité. Le dossier a été présenté au staff multidisciplinaire d'oncologie, il a été décidé 3 cures de chimiothérapie adjuvante à base de BEP comprenant bléomycine, étoposide et un sel de platine (cisplatinum).

## Discussion

Le tératome immature de l'ovaire est une tumeur maligne d'origine germinale, qui a été décrite pour la première fois en 1960 par Thürlbeck et Scully [1], contenant une quantité variable de tissu embryonnaire immature, généralement du tissu neuro-ectodermique [2]. Le tératome immature représente 3% des tératomes, 1% de tous les cancers ovariens et 20% des tumeurs malignes de l'ovaire d'origine germinale [3–5]. L'origine de ces tumeurs fait appel à plusieurs théories. Les travaux de Linder et al. ont initialement démontré que les tératomes provenaient d'une cellule germinale, unique, isolée dont l'anomalie survient dès la première division méiotique [6]. À partir de l'analyse des caryotypes de tératomes immatures ovariens, Ohama et al. ont confirmé cette première hypothèse, mais ont également montré que pour un certain nombre de tumeurs, l'anomalie survenait plus tardivement au cours de la deuxième division méiotique, voire à partir d'un ovocyte mature [7]. Sur le plan clinique l'âge moyen de survenue est de 19 ans [8], et touche préférentiellement l'enfant et l'adolescente. C'est le cas de notre patiente qui était âgée de 25 ans. Il n y a pas de signes spécifiques pouvant faire suspecter un tératome ovarien, la symptomatologie est très variables, comme toute masse ovarienne, allant d'un simple trouble des règles a des douleurs pelviennes, et c'est le cas chez notre patiente, on peut avoir aussi un trouble de transit ou même une masse abdomino-pelvienne, et parfois une complication révélatrice type compression, torsion, hémorragie, rupture, infection ou une ascite. L'examen clinique est aussi très variable allant d'un examen normal a une sensibilité latéro- utérine ou une masse abdomino-pelvienne. Dans notre cas l'examen avait objectivé une sensibilité latéro-utérine gauche, avec perception d'une masse a travers le cul de sac postérieur au toucher vaginal. L'aspect échographique des tératomes immatures est peu spécifique [9], les images en tomodensitométrie et résonance magnétique sont plus caractéristiques. Généralement sur le plan scanographique les tératomes immatures sont rarement kystiques et ils se présentent sous la forme d'une large tumeur irrégulière d'aspect mixte, tissulaire et graisseux, la partie solide étant constituée de nombreuses calcifications grossières et amorphes associées à des ilots graisseux disséminés et quelques rares microkystes [10], cette masse se rehausse après injection du produit de contraste. A l'IRM ils se présentent sous la forme d'une volumineuse portion tissulaire présentant quelques plages graisseuses peu abondantes, visibles en hypersignal T1, avec la présence de multiples microkystes au sein de la masse. Ainsi chez notre patiente l'échographie pelvienne a objectivée un utérus de taille normale avec en sus utérin une image solido-kystique avec cloison, confirmée sur les images scanographiques, par la mise en évidence d'une masse pelvienne solidokystique mesurant 110/80 mm, prolabée dans le cul de sac de douglas, rehaussée âpres injection du produit de contraste de façon hétérogène évoquant une masse ovarienne suspecte de malignité, ce qui rejoins les résultats de la littérature. Toute fois les études ont montrés que les examens radiologiques ne permettent pas de préjuger du grade histologique car le tératome immature de l'ovaire est une tumeur maligne composée de tissus dérivés des trois lignées cellulaires embryonnaires (le mésoderme, l'endoderme et l'ectoderme) différenciées en tissu neural, cartilage, et mésenchyme, à des stades de maturation différents, au sein de la tumeur. Leur potentiel malin est directement dépendant du degré d'immaturité et de la présence de neuroectoderme ce qui permet d'établir une classification en trois grades de malignité croissante selon la classification de Norris, cette classification a été proposée initialement par Thurlbeck et Scully [1] et modifiée en 1976 par Norris et O'Connor afin de mieux définir les indications thérapeutiques [11] (Tableau 1). Actuellement, cette classification tend à être simplifiée en bas et haut grade (Tableau 2). Dans notre cas les prélèvements réalises au niveau de la masse ont objectivés une prolifération de tissu neuroectodermique immature sous la forme de rosettes primitives neuroépithéliales, de tubes et de tissu cellulaire glial immature. Le tissu neural immature occupe largement plus de 5 champs au faible grossissement. L'existence d'éléments vitellins au sein du tératome immature a été rapportée comme une source de sécrétion anormale d'alphafoetoprotéine, en particulier chez les patientes qui présentent les taux les plus élevés. La présence de ces structures vitellines apparaît comme un facteur prédictif du risque de récidive [12]. L'alphafoetoprotéine peut être augmentée dans 18 à 45% des cas [12, 13]. Un taux sérique supérieur à 400 ng est considéré comme un facteur de risque d'évolution péjorative. Dans notre cas, l'alphafoetoprotéine n'a pas été demandée avant le traitement chirurgical.

**Tableau 1:** Grading des tératomes ovariens immatures de Norris et O’Connor (en 3 grades)

**Grade 0**	Tissu totalement mature activité mitotique rare
**Grade 1**	Tumeur contenant de rares foyers de tissu immature neuro-épithélial qui occupent moins d’un champ par lame au grossissement (40x)
**Grade 2**	Tumeur contenant de rares foyers de tissu immature neuro-épithélial qui occupent 1 à 3 champs par lame au grossissement (40x)
**Grade 3**	Tumeur contenant de larges plages de tissu immature neuro-épithélial qui occupent plus que 3 champs par lame au grossissement (40x)

**Tableau 2:** Grading des tératomes ovariens immatures en haut grade/bas grade et leur correspondance

	Classification en Haut grade/ Bas grade	Stade FIGO
**Tumeur ovarienne grade 1**	Bas grade	Ia
**Tumeur ovarienne grade 2 ou 3**	Haut grade	Ia
**Implants grade 2 ou 3**	Haut grade	>II
**Grade 0 implants péritonéaux** **Indépendamment du grade de la tumeur ovarienne**		>II

L'évolution des tératomes immatures est marquée par une croissance tumorale très rapide. L'extension est ensuite principalement locorégionale, responsable d'une invasion péritonéale, nécessitant une brèche capsulaire. Les lésions secondaires des tératomes immatures sont décrites sous la forme de granulations superficielles, fermes et de couleur grisâtre ou jaunâtre que l'on peut retrouver sur l'ensemble du péritoine abdominopelvien, parfois sur le grand épiploon ou d'autres organes comme le foie. Ces métastases sont généralement sous une forme immature. L'évolution propre des implants est mal connue. Ils peuvent rester asymptomatiques et stables, insensibles à la chimiothérapie ou subir une régression fibreuse. La dégénérescence gliale ou tératomateuse est exceptionnelle. Sur le plan thérapeutique et selon la société française de cancérologie 2013 le traitement des tératomes immatures est divisé en deux volets, un traitement chirurgical suivi par une chimiothérapie en fonction du grade histologique. Le traitement chirurgical est toujours conservateur et vise 3 objectifs: diagnostique (histologie), thérapeutique (ablation de la tumeur), et la détermination du stade d'extension, ainsi on recommande une chirurgie initiale au cours de laquelle on réalise au minimum une annexectomie unilatérale, une exploration complète du pelvis et de toute la cavité abdominale, un lavage péritonéal et/ou prélèvement de toute ascite, des biopsies péritonéales multiples systématiques y compris au niveau de l'épiploon et le prélèvement de tout élément suspect. Pour le curage ganglionnaire il n y a pas d'indication à un curage systématique pelvien et lombo-aortique en l'absence d'anomalie ganglionnaire, et un prélèvement sera réalisé en cas d'anomalie visible sur le scanner ou palpable lors de l'exploration chirurgicale. Pour l'ovaire controlatérale une inspection soigneuse s'impose, et une biopsie est recommandé par certains auteurs, il n y a pas d'indication a une annexectomie bilatérale systématique. Pour l'utérus pas de place a une hystérectomie [14, 15]. Le but de ce traitement conservateur est dans un souci de conservation maximale de la fertilité. Dans notre cas La patiente a bénéficiée initialement d'une laparotomie avec a l'exploration présence d'une masse ovarienne de 11 cm de diamètre, d'où la réalisation d'une annexectomie gauche avec biopsies multiples (Gouttières parieto-coliques droite et gauche, l'espace vésico-uterin, grand epiploon et le douglas), dont le résultat anatomo-pathologique était un tératome ovarien immature grade 3, avec un tissue graisseux sensiblement normal dans les différentes biopsies réalisés. Après le résultat anatomopathologique, le geste chirurgical a été complété par la réalisation d'une hystérectomie avec omentectomie et curage pelvien dont le résultat anatomo-pathologique était sans particularité. Notre attitude radicale est expliquée par le faite qu'un grand nombre de nos patientes sont perdue de vue juste après le geste chirurgicale et qu'on trouve des difficultés à les contacter pour compléter leur traitement. L'examen anatomopathologique de la pièce opératoire permet de confirmer le diagnostic, et de déterminer le grade qui conditionne un éventuel traitement adjuvant. Pour les tumeurs de grade 1, la surveillance clinique semble suffisante après traitement chirurgical conservateur seul. Cette attitude est confirmée par Carinelli sur une vaste série de 245 tératomes immatures de l'ovaire qui montre l'absence de récidive à long terme dans ce sous-groupe de tératome immature de l'ovaire après annexectomie unilatérale [16]. En cas de tératome immature de grade 2 ou 3, l'attitude à adopter, chimiothérapie adjuvante ou chirurgie de stadification, reste controversée. La chimiothérapie adjuvante est habituellement réservée aux tumeurs de grade 3, voire de grade 2. Le protocole le plus utilisé comporte trois molécules empruntées aux chimiothérapies du cancer testiculaire: BEP comprenant bléomycine, étoposide et un sel de platine (cisplatinum) [15]. Notre patiente a été mise sous le protocole BEP, et elle va recevoir trois cures de chimiothérapie. Selon Li et al., les progrès de la chimiothérapie et l'utilisation des sels de platine ont permis d'améliorer le pronostic des tératomes immatures dont la survie était de 40% avant 1983, alors qu'elle atteint 95% entre 1994 et 1998 [12]. Le pronostic des tératomes immatures reste quand même meilleur que celui des cancers épithéliaux de l'ovaire, et il est corrélé au grade de la tumeur primitive [2]. Les tumeurs de grade 1 restent de bon pronostic car le taux de survie à cinq ans est évalué entre 81 et 94% dans la littérature [2, 12]. Les tératomes immatures de grade 3 ont un potentiel hautement malin et leur évolution rapide, locale et à distance, est responsable de taux de récurrence et de décès plus élevés [2, 12, 17], avec un taux de survie a 5 ans de 90 a 100% avec chimiothérapie [18].

## Conclusion

Le tératome immature est une tumeur maligne dont le pronostic est directement corrélé au grade histologique. C'est une tumeur de la femme jeune, dont le diagnostic est suspecté par les examens radiologiques et confirmé par l'anatomo-pathologie. Sa prise en charge doit s'orienter le plus souvent possible vers un traitement chirurgical conservateur avec préservation de la fertilité, complété par une chimiothérapie en fonction du grade. La stratégie thérapeutique nécessite une prise en charge multidisciplinaire entre oncologues, gynécologues et anatomopathologistes.

## Conflits d’intérêts

Les auteurs ne déclarent aucun conflit d'intérêts.
